# Incidence of Side Effects Associated With Acetaminophen in People Aged 65 Years or More: A Prospective Cohort Study Using Data From the Clinical Practice Research Datalink

**DOI:** 10.1002/acr.25471

**Published:** 2024-12-25

**Authors:** Jaspreet Kaur, Georgina Nakafero, Abhishek Abhishek, Christian Mallen, Michael Doherty, Weiya Zhang

**Affiliations:** ^1^ University of Nottingham Nottingham United Kingdom; ^2^ University of Nottingham and National Institute for Health and Care Research Biological Research Centre Nottingham United Kingdom; ^3^ Keele University Keele United Kingdom; ^4^ University of Nottingham and Pain Centre Versus Arthritis Nottingham United Kingdom

## Abstract

**Objective:**

The main objective of this study is to examine the safety of oral acetaminophen at its therapeutic dose in adults aged ≥65 years.

**Methods:**

This population‐based cohort study used the Clinical Practice Research Datalink‐Gold data. Participants were aged ≥65 years registered with a UK general practice for at least 12 months between 1998 and 2018. Acetaminophen exposure was defined as at least two acetaminophen prescriptions within six months of the first acetaminophen prescription, the first prescription date being the index date. Acetaminophen nonexposure was defined as the absence of two acetaminophen prescriptions within six months over the study period. We calculated propensity score (PS) for acetaminophen prescription and undertook inverse probability treatment weighting using PS and PS‐matched analyses to account for confounding. Missing data were handled using multiple imputation. The adjusted hazard ratio (aHR) and 95% confidence interval (95% CI) were calculated using the Cox proportional hazards regression model.

**Results:**

In total, 180,483 acetaminophen exposed and 402,478 unexposed participants were included in this study. Acetaminophen exposure was associated with an increased risk of perforation or ulceration or bleeding (aHR 1.24; 95% CI 1.16–1.34), uncomplicated peptic ulcers (aHR 1.20; 95% CI 1.10–1.31), lower gastrointestinal bleeding (aHR 1.36; 95% CI 1.29–1.46), heart failure (aHR 1.09; 95% CI 1.06–1.13), hypertension (aHR 1.07; 95% CI 1.04–1.11), and chronic kidney disease (aHR 1.19; 95% CI 1.13–1.24).

**Conclusion:**

Despite its perceived safety, acetaminophen is associated with several serious complications. Given its minimal analgesic effectiveness, acetaminophen as the first‐line oral analgesic option for long‐term conditions in older people requires careful reconsideration.

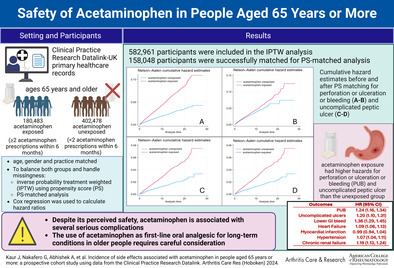

## INTRODUCTION

Almost all clinical guidelines advocate acetaminophen as the first‐line oral pharmacological treatment for pain due to osteoarthritis (OA), mainly because of its perceived safety over other oral analgesics.^1^, ^2^, ^3^, ^4^ However, recent studies have raised concerns that acetaminophen may be not as safe as previously thought.^5^, ^6^, ^7^, ^8^, ^9^

**SIGNIFICANCE & INNOVATIONS**

- Acetaminophen is a relatively weak analgesic, but largely because of its perceived safety, it has been recommended as the first‐line oral analgesic, especially in older people. However, this study shows a significant incidence of gastrointestinal, cardiovascular, and renal side effects in older people, who are prescribed acetaminophen repeatedly in the United Kingdom.
- Although the incidence may be lower, the side effect profile aligns with that of nonsteroidal anti‐inflammatory drugs and cyclooxygenase 2 (COX‐2) inhibitors, which reflects the now‐recognized COX inhibitory effect of acetaminophen.
- These findings support reconsideration of acetaminophen as an oral analgesic by guideline development groups who currently recommend its repeated prescription for long‐term conditions such as osteoarthritis.

Acetaminophen can cause cyclooxygenase (COX)–dependent side effects analogous to those of nonsteroidal anti‐inflammatory drugs (NSAIDs).^5^, ^10^, ^11^ The National Institute for Health and Care Excellence (NICE) in the UK highlighted this concern over the safety of acetaminophen in the 2014,^1^ updated guidance on management of OA and no longer recommends acetaminophen as a regular treatment in the 2022 update.^14^ This is related to both its nonclinically meaningful benefit, which had been confirmed earlier in a meta‐analysis (MA) and network MA,^12^, ^13^ and its potential harms.^14^

Ideally, randomized controlled trials (RCTs) are required to provide evidence of drug efficacy and safety. However, RCTs are not an optimal design to evaluate the safety of acetaminophen because of ethical concerns and cost implications that preclude the recruitment of enough people in an adequately powered study of sufficient duration. Therefore, the evidence concerning the safety of acetaminophen at its therapeutic dose primarily comes from postmarketing observational studies.^5^, ^8^, ^10^, ^15^, ^16^ Previous observational studies on acetaminophen have encountered methodologic challenges, such as channeling bias. This bias occurs when individuals, particularly older adults at higher risk of gastrointestinal and cardiovascular adverse events, are less likely to be given NSAIDs but are more likely to receive acetaminophen.^5^

A previous study that used propensity score (PS) matching, in which the researchers compared the safety profiles of topical NSAIDs with acetaminophen and oral NSAIDs in people with knee or hip OA, revealed that topical NSAIDs exhibited a better safety profile compared with acetaminophen or oral NSAIDS.^17^ However, that study did not include a comparison with no analgesics. It is still unclear whether individuals prescribed acetaminophen are at increased risk of developing gastrointestinal, cardiovascular, and renal adverse events compared with those not taking any analgesics.

To address this gap and mitigate channeling bias, we conducted this cohort study comparing acetaminophen exposure versus non‐exposure to any analgesics for major adverse events in the general population, as well as in people with OA as a subgroup analysis using the large UK Clinical Practice Research Datalink. The objectives of the study were 1) to examine the incidence of perforation or ulceration or bleeding (PUB), uncomplicated peptic ulcers, lower gastrointestinal (GI) bleeding, heart failure, myocardial infarction, hypertension, and chronic renal failure in people prescribed acetaminophen in the general population compared with people unexposed to analgesics; and 2) to examine the dose‐response relationship between acetaminophen prescription and specific adverse events.

## PATIENTS AND METHODS

### Study design

This was a population‐based cohort study, conducted with data from the Clinical Practice Research Datalink (CPRD) GOLD,^18^ where acetamiophen exposed participants were compared to acetaminophen unexposed for the incidence of major adverse events, including GI, cardiovascular (CV), and renal adverse events. The CPRD is one of the largest health care databases and has been demonstrated to be a reliable resource for research.^19^, ^20^, ^21^ It had collected anonymized patient data from 736 general practices, covering 17 million UK residents as of January 2018. Data were entered electronically either during a consultation with a general practitioner or through communication with other health professionals. Details of the patient demographics, medications, and diagnoses were recorded.^22^ The CPRD broadly represents the UK population in terms of age, gender, ethnicity, and body mass index (BMI).^23^ Although not explicitly designed for surveys or studies, such standard health data are cost‐effective for research purposes, allowing for examination of the effectiveness and safety of interventions in the real world.^22^

### Participants

In the United Kingdom, people aged ≥65 years are eligible for free prescriptions from their general practitioner, which permitted us to examine the safety of acetaminophen in older people in the United Kingdom. We included older adults aged ≥65 years at index date—the date of the first acetaminophen prescription between January 1, 1998, and January 1, 2018, who had been registered for at least 12 months with a general practice. The practices included were deemed up to standard by CPRD, i.e, contributing comprehensive, continuous, and complete high‐quality data for research purposes. We excluded participants with a diagnosis of the outcome of interest before the start of follow‐up (details given in exposure section below). Consent from individuals involved in this study was not sought because this was database research. The Independent Scientific Advisory Committee approved this cohort study with the protocol reference 19_131R.

### Exposure

Participants who were issued at least two acetaminophen prescriptions within six months, and not in combination with other analgesics such as codeine, were defined as exposed to acetaminophen to exclude occasional oral intake of acetaminophen for other acute conditions such as headache and influenza. The date of the first of the two prescriptions was assigned as the index date. All the records were obtained using the product codes (Supplementary Table S1). To avoid prevalent cases, we included only those who did not have an acetaminophen prescription in the 12 months before the index date.^24^ A landmark analysis was applied as a simple approach to minimize immortal time bias—a potential confounding that could influence the results when follow‐up is delayed one way or another.^25^ Participants were followed from the landmark date (i.e, the date 12 months after the index date) to avoid the chance of counting outcomes during the exposure period. This would also give a 12‐month exposure window from the index date to the landmark date to accumulate repeated acetaminophen doses for the dose‐response analysis. The follow‐up stopped at the earliest event, study end date (January 1, 2018), date of death, or transfer out of practices, whichever came first.

### Comparators

The control group (unexposed) included participants aged ≥65 years with less than two acetaminophen prescriptions within six months during the study period when exposure could be accrued. The controls were individually matched to acetaminophen exposed participants by year of birth, sex, and general practice on a 1:n (as available) basis, then further matched by PS on a 1:1 basis. The controls were assigned the index date and landmark date of their matched acetaminophen‐exposed participant.

### Outcomes

The outcomes of interest were the incident diagnosis of 1) GI conditions, specifically PUB, uncomplicated peptic ulcers (ulcers without bleeding or perforation), and lower GI bleeding; 2) CV outcomes, specifically hypertension, myocardial infarction, or heart failure; and 3) chronic renal failure. Patients with these outcomes were identified using relevant Read codes or Med codes recorded in the CPRD records. These Med codes were similar to those used in other studies to extract data on GI, CV, and renal events and were updated using the medical dictionary of the CPRD data set.^5^, ^21^

### Covariates

We shortlisted potential covariates such as age, sex, Charlson comorbidity index (CCI) (Supplementary Material: Covariates), opioids, NSAIDS, coxibs, aspirin, H2‐receptor blockers, proton pump inhibitors, dipyridamole, clopidogrel and lifestyle factors (smoking, alcohol, and BMI) based on their association with acetaminophen and the outcome of interest. These covariates were used to calculate the PS for acetaminophen prescription to account for confounding.^26^ Codes for the comorbidities were obtained from the Primary Care Unit, University of Cambridge (https://www.phpc.cam.ac.uk/pcu/cprd_cam/codelists/v11/) and updated using the CPRD code browser. We excluded any comorbidity from the CCI if it was an outcome to avoid including prevalent cases (Supplementary Table S2).

### Statistical analysis

Logistic regression was used to calculate the PS for acetaminophen prescription.^27^ Standardized differences (*d*) were used to examine the covariate balance between the exposed and unexposed participants. Covariates for which there was an imbalance, defined as *d* >0.1, were included as additional covariates in the subsequent Cox regression model.^28^ The kernel‐density plot was used to check the covariate balance graphically. The cumulative survival probability was estimated using the Kaplan‐Meier (Nelson‐Aalen) survival curve.^29^ The proportional hazards assumption was assessed using log‐log plots and the Schoenfeld residuals.^30^ The Cox regression model calculated the hazard ratio (HR) between the acetaminophen‐exposed versus nonexposed participants. Different Cox regression models were constructed: Model 1—age, sex, and general practice (GP) matched; Model 2—age, sex, GP, and PS matched; Model 3—age, sex, GP, and PS‐matched analyses adjusted for unbalanced covariates; and Model 4—inverse probability treatment weighting (IPTW) using PS. IPTW using PS was considered the main model given its potential to yield more precise estimates in time to event analyses than the PS‐matched method.^31^

We conducted a subgroup analysis restricted to participants with OA, a common condition of older people that is often associated with long‐term analgesic regime. In this subgroup analysis, the PS model was recalculated. This ensured that the matched pairs were comparable in this subgroup of people with OA and allowed us to capture the safety profile of acetaminophen in a common long‐term condition that often requires regular oral administration of acetaminophen. Statistical significance was considered at *P* < 0.05.

To assess a dose‐response relationship between acetaminophen and the outcomes, we calculated the number of acetaminophen prescriptions between index and landmark date. These were categorized as no prescription (reference group), one to two prescriptions, three to four prescriptions, five to six prescriptions, seven to eight prescriptions, and nine or more. To minimize channeling bias, the dose‐response analyses were repeated with the individuals prescribed one to two prescriptions as the reference category.

Missing data on BMI, alcohol use, and smoking were handled as a separate category in the PS‐matched analyses and by multiple imputation in the IPTW using PS analyses (Supplementary Material: Handling missing data). The data used in this study could be obtained directly from the CPRD upon request because of CPRD licensing rules.

## RESULTS

### Cohort description

Of 2,789,347 participants (exposure, 697,362 and nonexposure, 2,091,985) matched by age, sex, and practice, 582,961 were included in the IPTW using PS analyses, and 158,048 were successfully PS matched (79,024 acetaminophen exposed, 79,024 unexposed) (Supplementary Figure S1). The mean age ± SD of participants included in the IPTW using PS and PS‐matched cohort was 74.88 ± 7.29 years and 75.42 ± 7.51 years, respectively. Most participants self‐identified as female (Table 1). The mean ± SD duration of follow‐up was 1 ± 4.62 years.

**Table 1:** Participant characteristics*

	IPTW using PS sample (n = 582,961)^a^			PS‐matched sample (n = 158,048)^a^		
	Acetaminophen exposed	Acetaminophen unexposed	*d*	Acetaminophen exposed	Acetaminophen unexposed	*d*
n	180,483	402,478		79,024	79,024	
Continuous variables, mean ± SD						
Age	74.88 ± 7.29	74.99 ± 7.47	−0.01	75.42 ± 7.51	75.41 ± 7.75	0.00
Charlson's comorbidity index	0.43 ± 1.24	0.24 ± 0.93	0.15	0.53 ± 1.45	0.36 ± 1.21	**0.13**
Categorical covariates, n (%)						
Female	114,066 (63.20)	242,175 (60.17)	0.06	48,167 (60.95)	50,421 (63.80)	0.06
OA	55,038 (30.49)	60,349 (14.99)	0.38	28,356 (35.88)	24,391 (30.86)	**0.11**
BMI						
Underweight	3,647 (2.02)	5,505 (1.37)	0.05	2,330 (2.95)	1,625 (2.06)	0.06
Overweight	43,402 (24.02)	73,328 (18.22)	0.14	22,776 (28.82)	20,024 (25.34)	0.08
Obese	22,163 (12.28)	29,131 (7.24)	0.17	12,363 (15.64)	9,761 (12.35)	0.09
Missing	64,148 (35.54)	208,224 (51.74)	−0.33	19,101 (24.17)	25,648 (32.45)	−0.18
Smoking status						
Current	15,138 (8.39)	30,110 (7.48)	0.03	8,875 (11.23)	8,237 (10.42)	0.03
Ex‐smoker	44,190 (24.48)	63,636 (15.81)	0.21	21,471 (27.17)	18,347 (23.21)	0.09
Missing	32,468 (17.99)	168,282 (41.81)	−0.54	9,208 (11.65)	14,680 (18.57)	−0.19
Alcohol						
Current drinkers	79,661 (44.14)	144,373 (35.87)	0.17	40,956 (51.82)	38,149 (48.27)	0.07
Past drinkers	4,448 (2.46)	4,767 (1.18)	0.10	2,180 (2.76)	1,493 (1.89)	0.06
Missing	57,999 (32.14)	198,151 (49.23)	−0.35	16,978 (21.48)	22,904 (28.98)	−0.17
Other medication						
Opioids	178,328 (69.87)	76,896 (30.13)	2.76	76,878 (97.27)	76,878 (97.27)	0.00
NSAIDS	55,559 (30.78)	48,061 (11.94)	0.47	26,635 (33.70)	24,385 (30.85)	0.06
Coxibs	10,077 (5.58)	5,965 (1.48)	0.22	5,208 (6.59)	3,621 (4.58)	0.09
Clopidogrel	10,183 (5.64)	7,658 (1.90)	0.20	4,073 (5.15)	2,983 (3.77)	0.07
Dipyridamole	5,255 (2.91)	4,947 (1.23)	0.12	2,771 (3.51)	1,835 (2.32)	0.07
H2‐receptor blockers	18,745 (10.39)	17,714 (4.40)	0.23	10,914 (13.81)	8,213 (10.39)	**0.10**
Proton pump inhibitors	63,710 (35.30)	49,621 (12.33)	0.56	25,060 (31.71)	22,250 (28.15)	0.07
Aspirin	72,064 (39.93)	78,523 (19.51)	0.46	31,257 (39.55)	26,428 (33.44)	**0.13**

* Significant values are in bold. BMI, body mass index; *d*, standardized difference; IPTW, inverse probability treatment weighting; NSAID, nonsteroidal anti‐inflammatory drug; OA, osteoarthritis; PS, propensity score.

^a^ After age, sex, and general practice matching.

**Figure 1:**
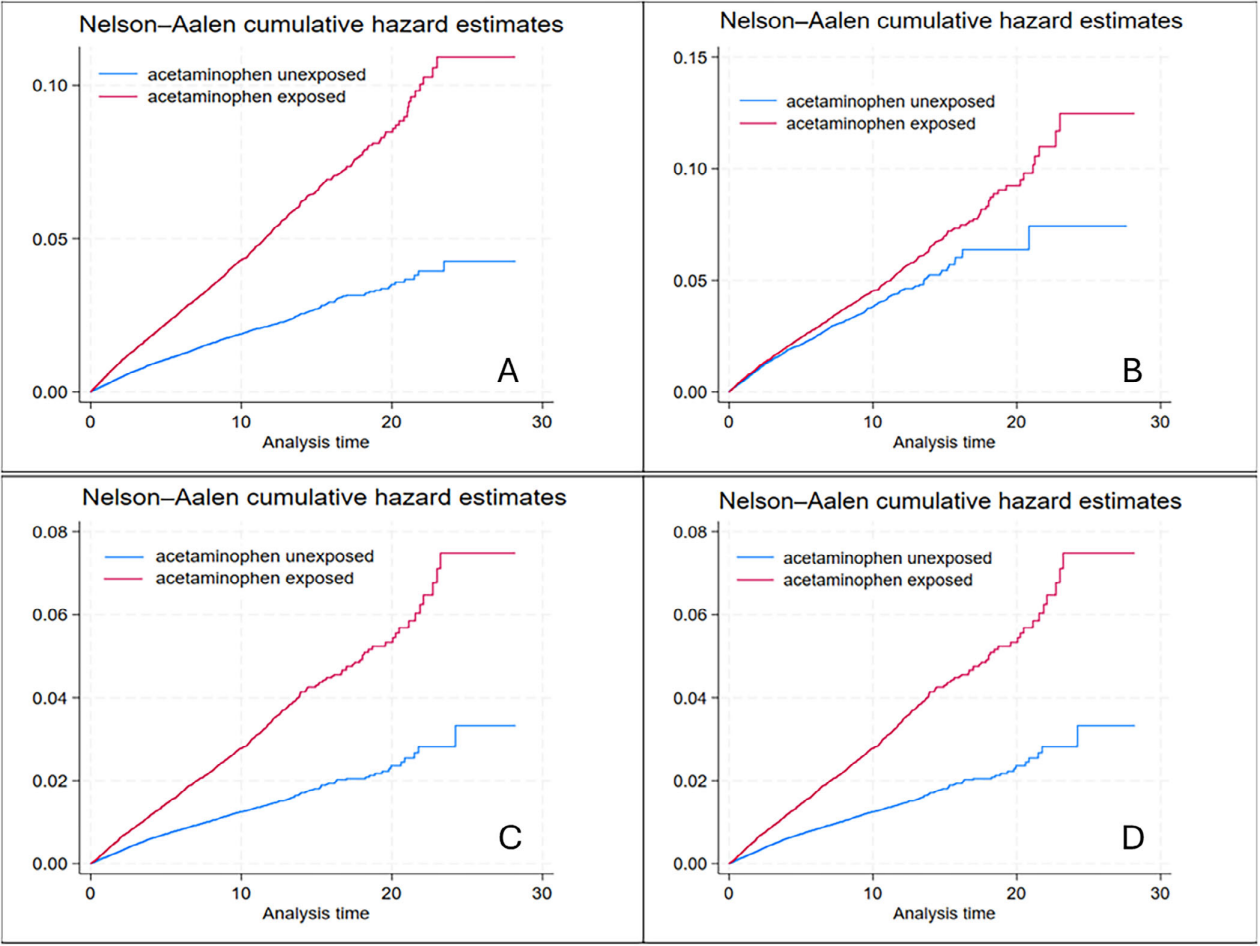
Cumulative hazard estimates before and after PS matching for perforation or ulceration or bleeding (A and B) and uncomplicated peptic ulcers (C and D) (after age, sex, and general practice matching). The red line represents that acetaminophen exposure has higher hazards for perforation or ulceration or bleeding and uncomplicated peptic ulcers than the unexposed group, represented by the blue line. PS, propensity score.

**Figure 2:**
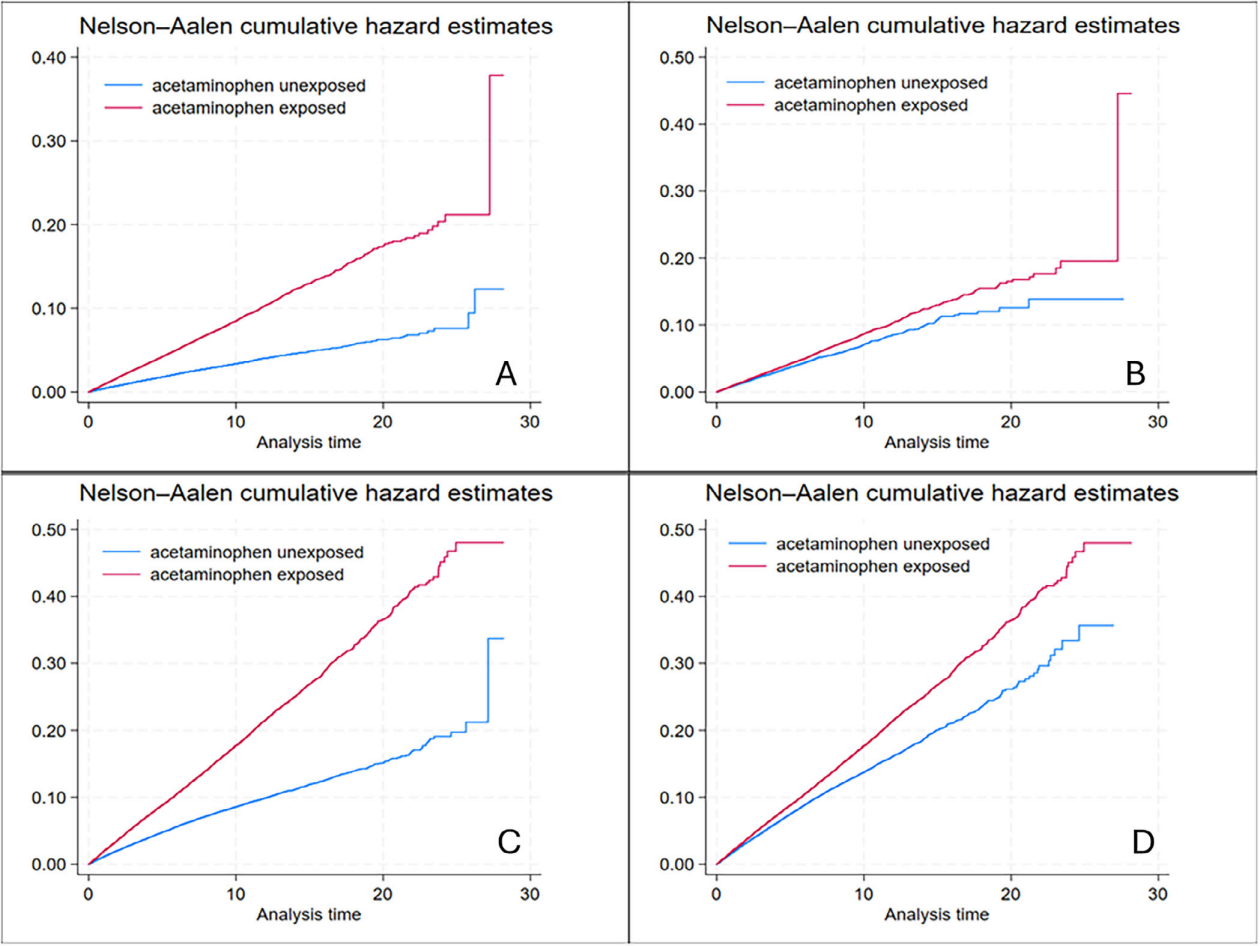
Cumulative hazard estimates for lower gastrointestinal bleeding and heart failure before PS matching (A and C, respectively) and after PS matching (B and D, respectively) (after age, sex, and general practice matching). The red line represents the acetaminophen exposure group and shows higher hazards for lower gastrointestinal bleeding and heart failure than the unexposed group, represented by the blue line. PS, propensity score. Color figure can be viewed in the online issue, which is available at http://onlinelibrary.wiley.com/doi/10.1002/acr.25471/abstract.

**Figure 3:**
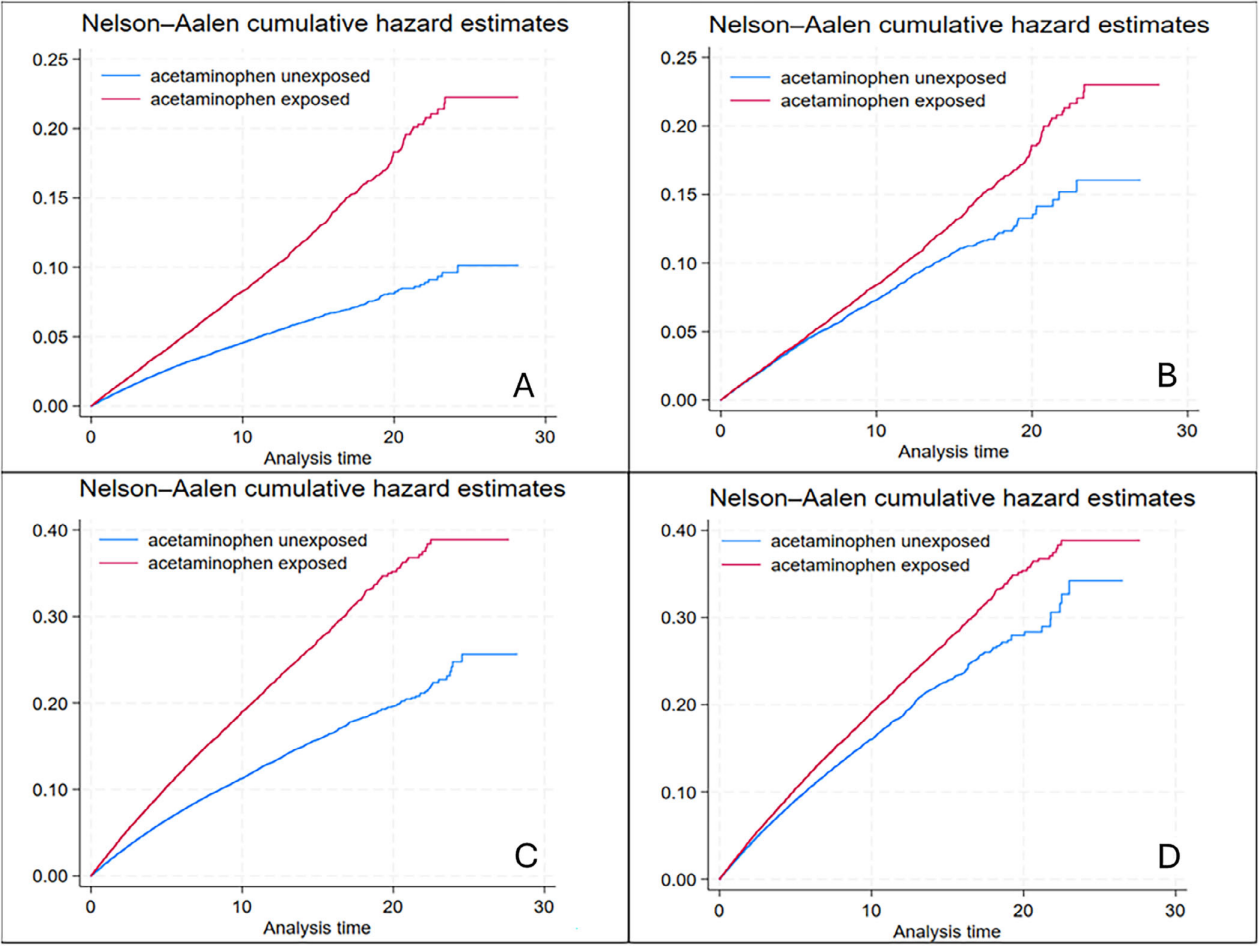
Cumulative hazard estimates for myocardial infarction and hypertension PS matching (A and C, respectively) and after PS matching (B and D, respectively) (after age, sex and general practice matching). The red line represents the acetaminophen exposure group and shows higher hazards for myocardial infarction and hypertension than the unexposed group, represented by the blue line. PS, propensity score. Color figure can be viewed in the online issue, which is available at http://onlinelibrary.wiley.com/doi/10.1002/acr.25471/abstract.

The Kaplan‐Meier curves for acetaminophen exposed compared with unexposed for PUB, uncomplicated peptic ulcers, lower GI bleeding, heart failure, myocardial infarction, hypertension, and chronic renal disease before PS matching are given in Figure 1A and C, Figure 2A and C, Figure 3A and C, and Supplementary Figure S2A, respectively. The results after PS matching are given in Figure 1B and D, Figure 2B and D, Figure 3B and D, and Supplementary Figure S2B, respectively. These estimates indicated that the cumulative hazards for PUB, uncomplicated peptic ulcers, lower GI bleeding, heart failure, myocardial infarction, hypertension, and chronic renal disease were higher in the acetaminophen‐exposed group compared with the unexposed group.

### Incidence of GI, CV, and renal events

There was an increased incidence of PUB, uncomplicated peptic ulcers, lower GI bleeding, heart failure, hypertension, and chronic renal failure in the acetaminophen‐exposed participants compared with the unexposed participants with adjusted HR (aHR) (95% confidence interval [95% CI]) after IPTW using PS of 1.24 (1.16–1.34), 1.20 (1.10–1.31), 1.36 (1.29–1.46), 1.09 (1.06–1.13), 1.07 (1.04–1.11), and 1.19 (1.13–1.24), respectively. Similar results were observed from the PS‐matched analyses (Table 2).

**Table 2:** HRs and 95% CIs for incidence of gastrointestinal, cardiovascular, and renal outcomes in the acetaminophen‐exposed group vs the nonexposed group*

Outcomes	Acetaminophen exposure	Event rate/1,000 person‐years (IPTW using PS sample)	Event rate/1,000 person‐years (PS‐matched sample)	Model 1, HR (95% CI)	Model 2, HR (95% CI)	Model 3, HR (95% CI)	Model 4, HR (95% CI)
PUB	No	5.51	11.54	**1.00**	**1.00**	**1.00**	**1.00**
	Yes	12.19	12.63	**2.21 (2.10–2.32)**	**1.10 (1.01–1.19)**	**1.06 (1.00–1.16)**	**1.24 (1.16–1.34)**
Uncomplicated peptic ulcers	No	3.65	7.72	**1.00**	**1.00**	**1.00**	**1.00**
	Yes	7.84	8.38	**2.15 (2.02–2.29)**	**1.09 (1.00–1.21)**	1.04 (0.95–1.16)	**1.20 (1.10–1.31)**
Lower GI bleed	No	9.53	19.87	**1.00**	**1.00**	**1.00**	**1.00**
	Yes	23.36	23.23	**2.45 (2.36–2.54)**	**1.20 (1.13–1.28)**	**1.15 (1.09–1.23)**	**1.36 (1.29–1.45)**
Heart failure	No	24.67	40.94	**1.00**	**1.00**		**1.00**
	Yes	48.97	49.52	**1.98 (1.93–2.03)**	**1.24 (1.20–1.27)**	NA	**1.09 (1.06–1.13)**
Myocardial infarction	No	13.23	23.00	**1.00**	**1.00**		**1.00**
	Yes	22.95	23.13	**1.73 (1.67–1.79)**	**1.04 (1.00–1.09)**	NA	0.99 (0.94–1.04)
Hypertension	No	33.20	50.16	**1.00**	**1.00**		**1.00**
	Yes	54.23	54.57	**1.62 (1.59–1.66)**	**1.10 (1.07–1.13)**	NA	**1.07 (1.04–1.11)**
Chronic renal failure	No	16.24	30.77	**1.00**	**1.00**		**1.00**
	Yes	37.08	40.90	**2.29 (2.25–2.33)**	**1.31 (1.27–1.35)**	**1.22 (1.18–1.25)**	**1.19 (1.13–1.24)**

* Significant results are in bold. Model 1, age, gender, and practice matched; model 2, age, gender, practice and PS matched; model 3, age, gender, practice and PS matched with further adjustments for the variables that were still not balanced after PS matching, ie, Charlson comorbidity index, OA, H2‐receptor blockers, aspirin for GI and Charlson comorbidity index, ex‐smokers for chronic renal failure; model 4, IPTW method using PS. GI, gastrointestinal; HR, hazard ratio; IPTW, inverse probability treatment weighting; NA, not applicable; OA, osteoarthritis; PS, propensity score; PUB, perforation or ulceration or bleeding; 95% CI, 95% confidence interval.

### Dose response

The association among developing PUB, uncomplicated peptic ulcers, and chronic renal failure increased with the number of acetaminophen prescriptions (Supplementary Table S3). A similar trend was observed when the analysis was restricted to the acetaminophen‐exposed–only group, with *P* for trend <0.01 (Supplementary Table S4).

### Subgroup analysis

There were 115,387 participants with OA, of whom 48,812 were matched on PS (24,406 acetaminophen exposed and 24,406 unexposed). In this population, exposure to acetaminophen was associated with increased incidence of lower GI bleeding, hypertension, and chronic renal failure with aHR (95% CI) 1.20 (1.09–1.33), 1.06 (1.00–1.13), and 1.15 (1.09–1.22), respectively (Table 3).

**Table 3:** Subgroup analysis in participants with OA*

Outcomes	Model 1, HR (95% CI)	Model 2, HR (95% CI)	Model 3, HR (95% CI)	Model 4, HR (95% CI)
PUB	**1.22 (1.10–1.35)**	1.08 (0.94–1.26)	1.07 (0.92–1.24)	1.13 (0.99–1.29)
Uncomplicated peptic ulcers	**1.15 (1.01–1.30)**	0.97 (0.81–1.16)	0.95 (0.79–1.14)	1.04 (0.89–1.22)
Lower GI bleed	**1.29 (1.19–1.39)**	1.09 (0.98–1.22)	1.05 (0.94–1.18)	**1.20 (1.09–1.33)**
Heart failure	**1.08 (1.03–1.14)**	**1.06 (1.00–1.11)**	**1.07 (1.02–1.13)**	0.98 (0.92–1.04)
Myocardial infarction	1.00 (0.93–1.07)	0.96 (0.90–1.03)	0.96 (0.88–1.03)	0.82 (0.75–1.04)
Hypertension	0.96 (0.91–1.01)	0.97 (0.93–1.02)	0.99 (0.94–1.05)	**1.06 (1.00–1.13)**
Chronic renal failure	**1.26 (1.22–1.31)**	**1.21 (1.15–1.28)**	**1.16 (1.10–1.22)**	**1.15 (1.09–1.22)**

* The incidence in the nonexposed group was used as the reference; significant results are in bold. Model 1, age, gender, and practice matched; model 2, age, gender, practice and PS matched; model 3, age, gender, practice and PS matched with further adjustments for the variables that were still not balanced after PS matching, ie, Charlson comorbidity index, OA, H2‐receptor blockers, aspirin for GI and Charlson comorbidity index, ex‐smokers for cardiovascular and renal events; model 4, inverse probability treatment weighting method using PS. GI, gastrointestinal; HR, hazard ratio; OA, osteoarthritis; PS, propensity score; PUB, perforation or ulceration or bleeding; 95% CI, 95% confidence interval.

## DISCUSSION

In this large study of 180,483 acetaminophen exposed participants and 402,478 unexposed participants aged 65 years and older in the UK primary care population, we found that acetaminophen exposure was associated with an increased incidence of PUB, uncomplicated peptic ulcers, lower GI bleed, heart failure, hypertension, and chronic renal failure. A dose‐response relationship was observed for PUB, uncomplicated peptic ulcers, and chronic renal failure. The robustness of these associations was supported by 1) the observed risk for PUB, uncomplicated peptic ulcers, and chronic renal failure across different models; 2) a similar dose‐response relationship when the analyses were restricted only to the acetaminophen‐exposed group; 3) an increased incidence of lower GI bleeding, hypertension, and chronic renal diseases observed in a subgroup analysis restricted to people with OA—a common long‐term painful condition often requiring regular analgesics aligned with that found in the overall cohort.

The findings of our study are consistent with previous observational studies that have reported an association between acetaminophen intake and the risk of GI complications and hypertension.^8^, ^15^, ^17^, ^32^, ^33^, ^34^, ^35^ According to an experimental study, acetaminophen exerts an inhibitory effect on peripheral COX enzymes, suggesting that it could be a possible mechanism for the GI bleeding associated with its prescription.^7^ Furthermore, the significant dose‐response relationship of an increased risk associated with acetaminophen exposure and PUB, uncomplicated ulcer, and chronic kidney disease according to the number of prescriptions also aligns with previous observational studies.^5^, ^16^, ^36^, ^37^

The majority of acetaminophen RCTs have not found any major adverse effects,^38^, ^39^, ^40^ apart from one that reported a drop in hemoglobin of ≥1 g/dL over 13 weeks, presumed to be due to GI bleeding, in 20% of participants with knee pain taking acetaminophen 3 g/day.^41^ This is because the RCTs were primarily designed for efficacy rather than adverse events, solely reported short‐term effects, were less powered, and recruited healthier and younger participants.

There is limited experimental evidence to support the impact of acetaminophen on GI, CV, or renal events. For example, prolonged acetaminophen ingestion might inhibit prostacyclin synthesis in humans, resulting in GI lesions and bleeding.^42^, ^43^, ^44^ Grèen et al^42^ suggested that acetaminophen could disadvantage people suffering from conditions in which prostacyclin‐mediated vascular defense mechanisms are pronounced, such as myocardial infarction, deep vein thrombosis, and after surgery. The reason could be that oral administration of 500 mg acetaminophen decreases urinary excretion of 2‐3‐dinor 6 keto prostaglandin F1alpha, a stable inactive metabolite of major endothelium‐derived COX‐2 prostacyclin,^42^ and can cause marked reduction in prostacyclin synthesis for a minimum of six to eight hours without affecting thromboxane production. Acetaminophen is a major metabolite of phenacetin, which has been associated with hepatotoxicity and renal damage, but the mechanism of renal toxicity due to acetaminophen is still debatable. Furthermore, Lorz et al^45^ suggested that acute tubular necrosis might be responsible for renal impairment in people taking acetaminophen long‐term. The tubular cells undergo endoplasmic reticulum (ER) stress preceding growth arrest and DNA damage–inducible protein 153 stimulation and alteration to the nucleus in addition to caspase‐12 cleavage. Therefore, acetaminophen may cause the induction of caspase‐mediated cell death, indicating its nephrotoxic potential, and ER stress could be recognized as a therapeutic target in nephrotoxicity.

The IPTW using PS and PS matching can only control the known confounding factors and cannot address unmeasured or unknown confounders. Therefore, our results are prone to potential confounding bias due to unmeasured/unknown confounders.^21^, ^46^

Only 27% of the study participants were included in the PS‐matched analyses, leading to a significant reduction in our study sample size. However, results from the PS‐matched analyses were consistent with those from the IPTW using PS, which used all study participants exposed to acetaminophen, providing internal validity of our findings and enhancing their generalizability to older people prescribed acetaminophen.

A significant caveat to the study is that there is no provision for recording over‐the‐counter prescriptions in the CPRD. This limitation was a reason to restrict the study to people aged ≥65 years, who were eligible for free prescriptions and therefore were less likely to purchase acetaminophen independently. Other reasons for selecting this age group include the fact that older people are at higher risk of GI, CV, and renal adverse events, making them more likely to be prescribed acetaminophen than oral NSAIDs and opioids. Despite this, over‐the‐counter (OTC) usage might have affected both the exposure and nonexposure groups. It was assumed that the distribution would be random for other OTC analgesics but slightly more toward the nonexposure group, as those prescribed acetaminophen may be less likely to purchase it independently. Therefore, any such imbalance was expected to underestimate rather than overestimate the HR. The findings might be more applicable to settings where acetaminophen is predominantly prescribed as a medication. In populations where OTC remedies are more common across ages, the observed associations might differ.

Furthermore, acetaminophen is often taken episodically and for multiple reasons, making it difficult to define people exposed to acetaminophen and those not exposed to acteminophen. In this study, minimal acetaminophen exposure was defined as two or more prescriptions within six months of the first acetaminophen prescription. However, the subsequent intake of acetaminophen over time was unknown. The immortal time bias could occur not only within the exposure window of the initial 12‐month period, but also throughout the entire follow‐up period. Therefore, a time‐varying exposure analysis is necessary to account for dynamic changes in oral consumption of acetaminophen, to allow the counting of events according to exposure and nonexposure after the landmark date, and to better reflect the episodic taking of acetaminophen and its association with adverse effects, such as GI bleeding.

This population‐based cohort study, in which the likelihood of confounding by indication was minimized using PS methods, has produced robust evidence concerning the safety of acetaminophen in older adults. Although the incidence of acetaminophen side effects may be lower than that of NSAIDs and COX‐2 inhibitors, their side effect profiles are similar, which reflects the now‐recognized COX inhibitory effect of acetaminophen.^39^ These data further challenge whether acetaminophen should be retained as the first‐line oral analgesic, especially in older people for common chronic painful conditions, given its nonclinically meaningful benefits and potential harms, and support the recent recommendation by NICE to not prescribe acetaminophen for OA.^14^ A study in which acetaminophen prescription is modeled as a time‐varying exposure should be undertaken to confirm these findings.

This study provides the most recent evidence regarding the risk of important adverse events associated with oral administration of acetaminophen in the general population, as well as people with OA aged ≥65 years. Given the low analgesic benefit of acetaminophen in OA and its potential harms, existing guidelines recommending acetaminophen as the first‐line oral drug treatment for OA require reassessment.

## AUTHOR CONTRIBUTIONS

All authors were involved in drafting the article or revising it critically for important intellectual content, and all authors approved the final version to be published. Dr Kaur had full access to all of the data in the study and takes responsibility for the integrity of the data and the accuracy of the data analysis. Zhang is the guarantor of the project.

### Study conception and design

Doherty, and Zhang.

### Acquisition of data

Kaur, Nakafero, and Abhishek.

### Analysis and interpretation of data

Kaur, Nakafero, Abhishek, Mallen, Doherty, and Zhang.

## Supporting information


Disclosure form

**Appendix S1:** Supplementary Information
