# Supplementary material for: Incidence of Side Effects Associated With Acetaminophen in People Aged 65 Years or More: A Prospective Cohort Study Using Data From the Clinical Practice Research Datalink
Source: Arthritis Care Res (Hoboken). 2024 Dec 25;77(5):666–75. doi: 10.1002/acr.25471 (PMC12038216; doi:10.1002/acr.25471)
Supplement: Supplementary file 2 — Appendix S1: Supplementary Information [file ACR-77-666-s002.docx]

# Supplementary

**Product Codes**

**Supplementary Table S1: Exposure variable acetaminophen (paracetamol)**

| prodcode | product name |
| --- | --- |
| 7 | Paracetamol 500mg Tablets |
| 139 | Paracetamol 500mg capsules |
| 258 | Panadol 500mg Tablets (GlaxoSmithKline Consumer Healthcare) |
| 1609 | Paracetamol 500mg soluble Tablets |
| 1862 | Paracetamol 500mg/5ml oral suspension sugar free |
| 2800 | Panadol 500mg Soluble Tablet (GlaxoSmithKline Consumer Healthcare) |
| 3313 | Calpol six plus 250mg/5ml Oral suspension sugar free (McNeil Products Ltd) |
| 4186 | Paracetamol 250mg/5ml oral suspension sugar free |
| 5239 | Paracetamol 1g oral powder sachets |
| 6571 | Paracetamol 240mg oral powder sachets sugar free |
| 7205 | Panadol 500mg capsules (GlaxoSmithKline Consumer Healthcare) |
| 9712 | Paracetamol 250mg or dispersible Tablets sugar free |
| 9914 | Calpol Six Plus Fast melts 250mg Tablets (McNeil Products Ltd) |
| 10748 | Tramil 500mg Capsule (Wyeth Consumer Healthcare) |
| 15238 | Paracetamol 500mg Tablets (Zentiva) |
| 18799 | Flu strength hot lemon 1g Powder (A H Pharmaceuticals Ltd) |
| 20068 | Paracetamol 250mg/5ml oral suspension sugar free |
| 20116 | Paradote 100mg/500mg Tablets (Sinclair IS Pharma Plc) |
| 22288 | Medinol 250mg/5ml Oral suspension (SSL International Plc) |
| 23716 | Paracetamol 500mg caplets (IVAX Pharmaceuticals UK Ltd) |
| 23840 | Panasorb 500mg Tablet (Sanofi-Synthelabo Ltd) |
| 24000 | Mandanol 500mg Tablets (M & A Pharmachem Ltd) |
| 24075 | Mandanol 500mg caplets (M & A Pharmachem Ltd) |
| 24534 | Anadin Paracetamol 500mg Tablets (Pfizer Consumer Healthcare Ltd) |
| 24947 | Co-methiamol 100mg/500mg Tablets |
| 25895 | Hedex 500mg Tablets (Omega Pharma Ltd) |
| 26988 | Paracets 500mg Tablet (Sussex Pharmaceutical Ltd) |
| 27452 | Paracetamol 500mg soluble Tablets (Zentiva) |
| 27459 | Paracetamol 500mg caplets (Zentiva) |
| 28211 | Paracetamol 250mg/5ml oral suspension sugar free |
| 28344 | Paracetamol 500mg caplets (Wockhardt UK Ltd) |
| 28346 | Paracetamol 500mg Tablet (M & A Pharmachem Ltd) |
| 28955 | Panadol ActiFast 500mg Tablets (GlaxoSmithKline Consumer Healthcare) |
| 31196 | Paracetamol 500mg Tablets (IVAX Pharmaceuticals UK Ltd) |
| 31257 | Paracetamol 500mg caplets (Galpharm International Ltd) |
| 32163 | Paracetamol 500mg Tablets (Vantage) |
| 32839 | Paracetamol 500mg Tablets (A H Pharmaceuticals Ltd) |
| 32970 | Paracets 500mg capsules (Sussex Pharmaceutical Ltd) |
| 32993 | Children's Lemsip Cold & Flu Blackcurrant oral powder sachets (Reckitt Benckiser Healthcare (UK) Ltd) |
| 33104 | Paraclear 500mg Soluble Tablet (Roche Consumer Health) |
| 33614 | Obimol 500mg Tablet (Ayrton Saunders Ltd) |
| 33666 | Paracetamol 500mg Tablets (Actavis UK Ltd) |
| 33710 | Paracetamol 500mg caplets (A H Pharmaceuticals Ltd) |
| 33826 | Paracetamol 500mg Tablet (Teva UK Ltd) |
| 34164 | Paracetamol 500mg soluble Tablets (Fannin UK Ltd) |
| 34209 | Paracetamol 500mg/5ml Oral suspension sugar free (Rosemont Pharmaceuticals Ltd) |
| 34305 | Paracetamol 500mg Tablet (M & A Pharmachem Ltd) |
| 34350 | Paracetamol 500mg Tablet (Celltech Pharma Europe Ltd) |
| 34396 | Paracetamol 500mg Tablet (Family Health) |
| 34409 | Paracetamol 500mg Tablet (Co-operative) |
| 34500 | Paracetamol 500mg Tablets (Kent Pharmaceuticals Ltd) |
| 34669 | Paracetamol 500mg capsules (Zentiva) |
| 34858 | Paracetamol Capsule (Co-operative) |
| 34954 | Paracetamol 500mg Tablets (Aspar Pharmaceuticals Ltd) |
| 35679 | Paracetamol 500mg Tablet (Nucare Plc) |
| 36754 | Paracetamol 500mg soluble Tablets (A H Pharmaceuticals Ltd) |
| 38032 | Paracetamol 500mg capsules (Teva UK Ltd) |
| 38984 | Paracetamol 500mg capsules (Actavis UK Ltd) |
| 39333 | Paracetamol 500mg Tablet (Aspar Pharmaceuticals Ltd) |
| 39481 | Panadol Advance 500mg Tablets (GlaxoSmithKline Consumer Healthcare) |
| 39934 | Paracetamol 500mg caplets (Almus Pharmaceuticals Ltd) |
| 39940 | Panadol ActiFast Soluble Tablets (GlaxoSmithKline Consumer Healthcare) |
| 40107 | Paracetamol 500mg soluble Tablets (Kent Pharmaceuticals Ltd) |
| 40158 | Paracetamol 500mg Capsule (A H Pharmaceuticals Ltd) |
| 42201 | Paracetamol 500mg capsules (A H Pharmaceuticals Ltd) |
| 42371 | Paracetamol 1g Tablets |
| 42514 | Paracetamol oral liquid |
| 42834 | Panadol OA 1000mg Tablets (GlaxoSmithKline Consumer Healthcare) |
| 43028 | Paracetamol 650mg oral powder sachets |
| 43233 | Paracetamol 500mg capsules (Kent Pharmaceuticals Ltd) |
| 43252 | Paracetamol 500mg capsules (Aspar Pharmaceuticals Ltd) |
| 43479 | Paracetamol 500mg caplets (Actavis UK Ltd) |
| 45259 | Paracetamol 500mg Tablet (OBG Pharmaceuticals Ltd) |
| 45298 | Paracetamol 500mg Tablets (Galpharm International Ltd) |
| 46544 | Paracetamol 500mg capsules (Wockhardt UK Ltd) |
| 47116 | Paracetamol 500mg soluble Tablets (Almus Pharmaceuticals Ltd) |
| 47211 | Paracetamol 500mg capsules (Focus Pharmaceuticals Ltd) |
| 47834 | Paracetamol 500mg soluble Tablets (Actavis UK Ltd) |
| 48535 | Paracetamol 500mg caplets (Rusco Ltd) |
| 48597 | Paracetamol 500mg caplets (Alliance Healthcare (Distribution) Ltd) |
| 49096 | Paracetamol 500mg caplets (Lloyds Pharmacy Ltd) |
| 49105 | Paracetamol 500mg caplets (Teva UK Ltd) |
| 49417 | Paracetamol 500mg caplets (Kent Pharmaceuticals Ltd) |
| 49575 | Paracetamol 500mg caplets (Vantage) |
| 49849 | Paracetamol 500mg caplets (Phoenix Healthcare Distribution Ltd) |
| 50482 | Paracetamol 500mg Tablets (DE Pharmaceuticals) |
| 50504 | Numark Paracetamol 500mg capsules (Numark Ltd) |
| 53952 | Paracetamol 500mg capsules (Lloyds Pharmacy Ltd) |
| 54179 | Paracetamol 500mg Tablets (The Boots Company Plc) |
| 55129 | Paracetamol 500mg caplets (Waymade Healthcare Plc) |
| 55418 | Paracetamol 500mg Tablet (Almus Pharmaceuticals Ltd) |
| 56046 | Paracetamol 500mg capsules (Waymade Healthcare Plc) |
| 56566 | Paracetamol 500mg caplets (J M McGill Ltd) |
| 56945 | Paracetamol 500mg soluble Tablets (Waymade Healthcare Plc) |
| 57650 | Paracetamol 500mg capsules (Sigma Pharmaceuticals Plc) |
| 58526 | Paracetamol 500mg caplets (AM Distributions (Yorkshire) Ltd) |
| 58582 | Paracetamol 500mg Tablets (Teva UK Ltd) |
| 58743 | Paracetamol 500mg Tablets (Accord Healthcare Ltd) |
| 59599 | Paracetamol 500mg Tablets (Wockhardt UK Ltd) |
| 60378 | Flu Strength Hot Lemon Powders 1g oral powder sachets (Bell, Sons & Co (Druggists) Ltd) |
| 60442 | Paracetamol 500mg Tablets (Alliance Healthcare (Distribution) Ltd) |
| 60812 | Paracetamol 500mg caplets (Ethigen Ltd) |
| 61124 | Paracetamol 500mg caplets (Bristol Laboratories Ltd) |
| 61522 | Paracetamol 500mg caplets (Numark Ltd) |
| 61963 | Paracetamol 500mg caplets (Ennogen Healthcare Ltd) |
| 62088 | Paracetamol 500mg caplets (Icarus Pharmaceuticals Ltd) |
| 62743 | Boots Paracetamol 500mg capsules (The Boots Company Plc) |
| 63543 | Paracetamol 500mg Tablets (Sigma Pharmaceuticals Plc) |
| 63943 | Paracetamol 500mg caplets (Crescent Pharma Ltd) |
| 63962 | Paracetamol 500mg soluble Tablets (Teva UK Ltd) |
| 65107 | Paracetamol 500mg capsules (Bristol Laboratories Ltd) |
| 65239 | Paracetamol 500mg caplets (Mawdsley-Brooks & Company Ltd) |
| 65299 | Paracetamol 500mg caplets (Sigma Pharmaceuticals Plc) |
| 65330 | Paracetamol 500mg capsules (DE Pharmaceuticals) |
| 66097 | Paracetamol 500mg capsules (Almus Pharmaceuticals Ltd) |
| 67086 | Paracetamol 500mg capsules (Galpharm International Ltd) |
| 67784 | Paracetamol 500mg capsules (Almus Pharmaceuticals Ltd) |
| 68070 | Paracetamol 500mg Tablets (Zanza Laboratories Ltd) |
| 70018 | Boots Paracetamol 500mg caplets (The Boots Company Plc) |
| 70326 | Paravict 500mg Tablets (Ecogen Europe Ltd) |
| 71270 | Paracetamol 500mg effervescent Tablets sugar free |
| 72131 | Paracetamol 500mg capsules (Mawdsley-Brooks & Company Ltd) |
| 72288 | Paracetamol 500mg caplets (Accord Healthcare Ltd) |
|  |  |

# Covariates

## Age and gender

Although the literature regarding the safety of acetaminophen with age and gender is sparse, these were considered significant potential confounding factors, as acetaminophen is a highly preferred analgesic over its contemporary medications, such as non-steroidal anti-inflammatory drugs (NSAIDs), in the older population.

## Charlson co-morbidity index (CCI)

The co-morbidity disease burden was measured and adapted using the Charlson co-morbidity index ^[1]^. It was first identified by Charlson in 1987 and is a validated weighted co-morbidity index. A weighted score was assigned to each comorbid condition based on Risk ratio (RR) of 1-year mortality to analyse the disease burden ^[1]^. The CCI considers myocardial infarction, congestive heart failure, cerebrovascular disease, chronic pulmonary disease, connective tissue disease, ulcer disease, liver disease, diabetes, renal disease, hemiplegia, an acquired immunodeficiency syndrome (AIDS) and cancer. A weighted index was developed and assigned based on the severity and number of comorbid conditions.

**Supplementary Table S2: List of co-morbidities included in Charlson Co-morbidity Index.**

| Co-morbidities | IPTW using PS sample (n= 582,961)^a^ | | | After PS-matching | |
| --- | --- | --- | --- | --- | --- |
|  | **Acetaminophen exposed** | **Acetaminophen unexposed** | **Acetaminophen exposed** | | **Acetaminophen unexposed** |
| Cerebrovascular | 33,443 (18.53) | 43,376 (10.78) | 15,049 (19.04) | | 12,719 (16.09) |
| Rheumatological conditions | 11,957 (6.63) | 12,692 (3.15) | 5,553 (7.03) | | 5,248 (6.64) |
| Peripheral vascular diseases | 10,736 (5.95) | 14,497 (3.60) | 5,278 (6.68) | | 5,227 (6.61) |
| Peptic ulcer* | 11,018 (6.10) | 14,187 (3.52) | 5,404 (6.84) | | 4,887 (6.18) |
| Metastatic cancer | 1,946 (1.08) | 2,874 (0.71) | 857 (1.08) | | 1,165 (1.47) |
| Moderate liver disease | 462 (0.26) | 685 (0.17) | 210 (0.27) | | 217 (0.27) |
| Mild liver disease | 226 (0.13) | 317 (0.08) | 105 (0.13) | | 107 (0.14) |
| Myocardial infarction* | 15,849 (73.74) | 22,431 (74.87) | 9,872 (74.79) | | 6,839 (65.55) |
| Hemiplegia | 1,240 (0.69) | 1,532 (0.38) | 540 (0.68) | | 472 (0.60) |
| Diabetes no complications | 20,627 (11.43) | 29,006 (7.21) | 9,462 (11.97) | | 8,670 (10.97) |
| Diabetes end complications | 2,998 (1.66) | 4,242 (1.05) | 1,393 (1.76) | | 1,328 (1.68) |
| COPD | 27,302 (15.13) | 43,924 (10.91) | 13,549 (17.14) | | 14,717 (18.62) |
| Congestive heart failure* | 40,567 (22.48) | 56,443 (14.02) | 20,673 (26.16) | | 17,790 (22.51) |
| Chronic kidney disease* | 21,803 (12.08) | 25,683 (6.38) | 9,531 (12.06) | | 8,069 (10.21) |
| Cancer | 23,608 (13.08) | 32,587 (8.10) | 10,635 (13.46) | | 10,787 (13.65) |
| AIDS | 6 (0.00) | 9 (0.00) | 2 (0.00) | | 4 (0.01) |
| Dementia | 20,406 (11.31) | 17,527 (4.35) | 6,791 (8.59) | | 4,483 (5.67) |

PS - propensity score, AIDS - Acquired Immuno Deficiency Syndrome, COPD – Chronic obstructive pulmonary disease, (*) was excluded from the Charlson Co-morbidity Index if it was an outcome. ^a^After age, sex and General Practice matching

## Medication

The other medications selected for each model included NSAIDs, coxibs, aspirin, opioids, H2-receptor blockers, proton pump inhibitors, dipyridamole and clopidogrel. Exposure to these drugs was defined if their prescription falls before the landmark date. These variables were dichotomised (0 = unexposed, 1 = exposed), and the latest record was retained for the analysis before entering this cohort.

## Lifestyle factors

The lifestyle factors such as body mass index (BMI), smoking and alcohol intake were also considered potential confounders—the records of BMI before the landmark date were recorded from additional data files. If BMI data was missing, it was calculated from weight and height records in the additional file. If BMI, height, and weight were all missing, they were coded as missing. The smoking and alcohol consumption were extracted using additional records data files before the landmark date. When there was more than one entry for smoking and alcohol status, the latest record before the landmark date was used for analysis.

# **Handling missing data**

We undertook two approaches to manage missing data for BMI, smoking and alcohol. Firstly, we conducted propensity score (PS) matching by treating the missingness of these features as a separate indicator category. PS matching also balanced the covariates in both groups (acetaminophen exposure and acetaminophen unexposed). The estimates from the PS matching dataset were described as Model 2, and in some instances, if the features were still unbalanced, further adjustments were carried out, and estimates were presented as Model 3 (gastrointestinal and renal). However, using PS matching, there was a greater possibility of dropping many participants from the analysis, leading to a reduced sample size ^[2]^. Therefore, we performed multiple imputations (MI) with chained equations to substitute the missing values for BMI (46.7%), smoking (34%), and alcohol intake (43.9%) using five imputations. The distribution of observed data before and after performing multiple imputations was compared using midiagplots. Each of the five imputed datasets was analysed separately, and then the estimates were pooled using Rubin's rules. Due to rare events, the inverse probability treatment weighting (IPTW) using PS was implemented to handle confounding by indication bias ^[2, 3]^. The IPTW was determined within each of the five imputed datasets for each participant. In the IPTW, for each study participant within the dataset, formal weights were assigned equal to the reciprocal of their probability of obtaining treatment in reality while taking into account their baseline covariates ^[4, 5]^. During the calculation of formal weights, logistic regression calculated the probability of receiving acetaminophen. The weights were further calculated from the predicted probability (PS of receiving acetaminophen as if the participant received treatment (status=1)). The calculated propensity score weight was 1/PS, and if there was no treatment received by the participant (status=0), then the formal weight was calculated as 1/(1-ps) ^[6]^. HR was calculated using COX regression and the results were presented as Model 4.


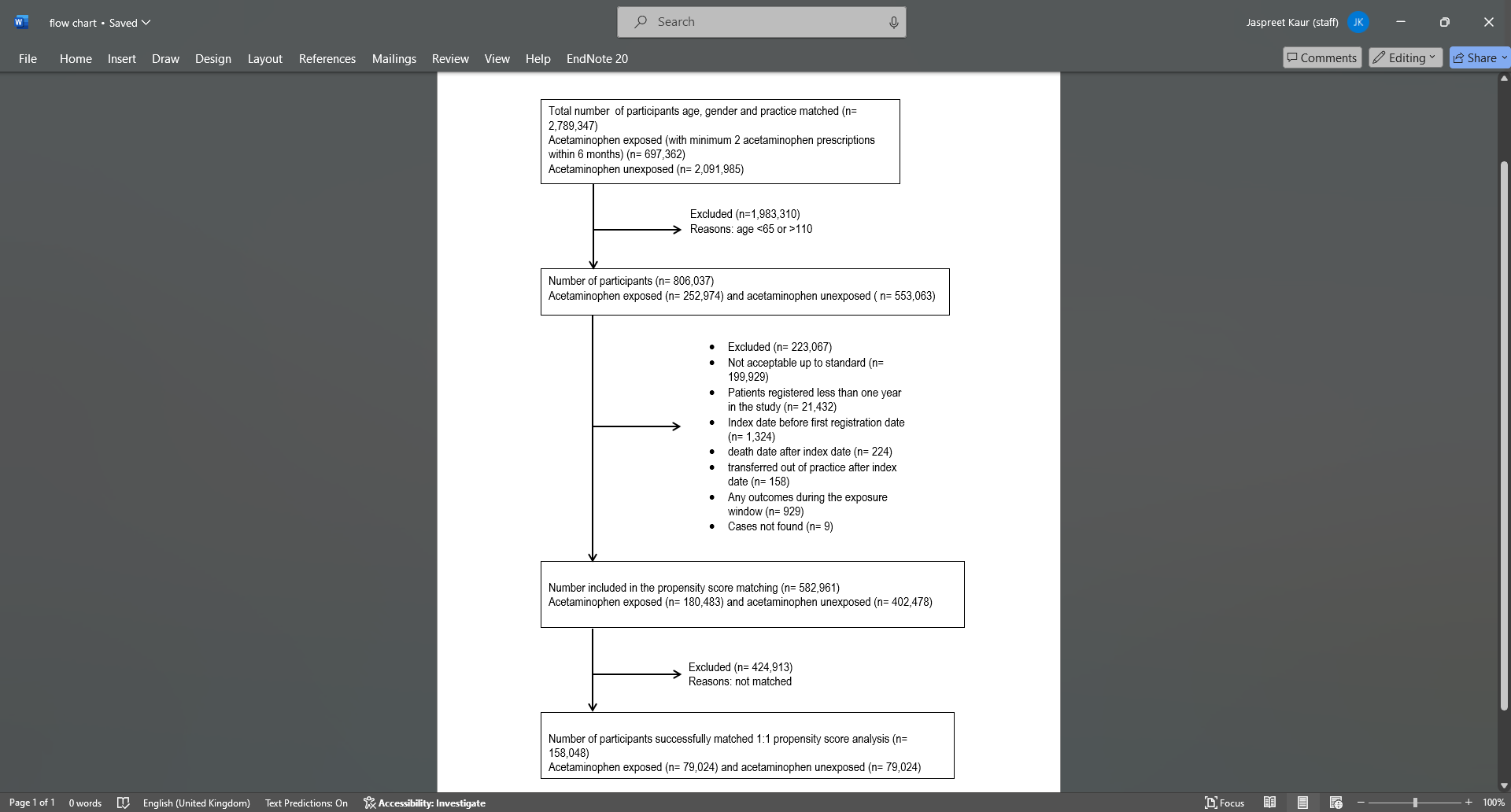


**Supplementary Figure S1: Participant selection process using propensity score matching.**

**
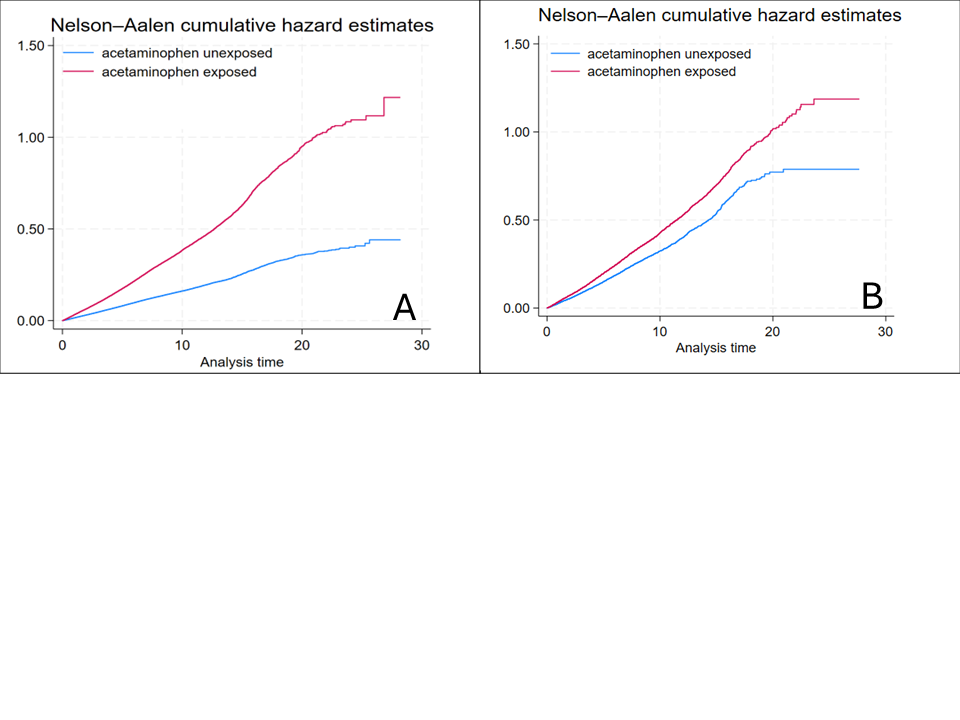
**

**Supplementary Figure S2: Cumulative hazards estimate for chronic renal failure in the acetaminophen exposed group (red lines) compared to the unexposed group (blue lines) before the PS-matching image (A) and after the PS-matching image (B) (after age, sex and General Practice matching). The red line represents the acetaminophen exposure group and shows higher hazards for chronic renal failure than the unexposed group, represented by the blue line.**

**Supplementary Table S3: Dose-response relationship between acetaminophen exposure and gastrointestinal, cardiovascular, renal outcomes using the inverse probability treatment weighting method with PS.**

| Number of prescriptions | PUB  HR (95%CI) | Uncomplicated ulcers  HR (95%CI) | Lower gastrointestinal bleed HR (95%CI) | Heart Failure HR (95%CI) | Myocardial infarction HR (95%CI) | Hypertension  HR (95%CI) | Chronic renal failure (95%CI) |
| --- | --- | --- | --- | --- | --- | --- | --- |
| Non-exposed | Reference | Reference | Reference | Reference | Reference | Reference | Reference |
| 1-2 | **1.15 (1.02, 1.30)** | 1.12 (0.97, 1.30) | **1.35 (1.14, 1.61)** | 1.01 (0.95, 1.07) | 0.94 (0.86, 1.03) | **1.08 (1.02, 1.14)** | **2.15 (2.08, 2.22)** |
| 3-4 | **1.17 (1.05, 1.29)** | **1.14 (1.00, 1.30)** | **1.34 (1.25, 1.44)** | 1.01 (0.96, 1.07) | 0.97 (0.90, 1.04) | **1.13 (1.08, 1.18)** | **2.18 (2.12, 2.25)** |
| 5-6 | **1.22 (1.08, 1.37)** | **1.15 (1.01, 1.33)** | **1.44 (1.32, 1.56)** | **1.13 (1.07, 1.21)** | 1.03 (0.94, 1.12) | **1.11 (1.05, 1.18)** | **2.36 (2.84, 2.44)** |
| 7-8 | **1.28 (1.11, 1.47)** | **1.20 (1.01, 1.42)** | **1.33 (1.21, 1.47)** | **1.10 (1.02, 1.19)** | 1.03 (0.93, 1.14) | **1.10 (1.03, 1.18)** | **2.54 (2.44, 2.63)** |
| 9 or more | **1.44 (1.29, 1.60)** | **1.39 (1.22, 1.58)** | **1.37 (1.26, 1.48)** | **1.25 (1.18, 1.32)** | 1.01 (0.93, 1.09) | 0.95 (0.90, 1.00) | **2.55 (2.48, 2.63)** |
|  | P = 0.00 | P = 0.00 | P = 0.00 | P = 0.01 | P = 0.05 | P = 0.07 | P = 0.00 |

PUB – perforation or ulceration or bleeding, HR – hazard ratio, CI – confidence interval, P – P_trend_, and significant results are in bold.

**Supplementary Table S4: Dose-response relationship between acetaminophen exposure and gastrointestinal, cardiovascular, and renal outcomes when restricted to exposure only using the inverse probability treatment weighting method with PS.**

| Number of prescriptions | PUB  HR (95%CI) | Uncomplicated ulcers  HR (95%CI) | Lower gastrointestinal bleed HR (95%CI) | Heart Failure HR (95%CI) | Myocardial infarction HR (95%CI) | Hypertension  HR (95%CI) | Chronic renal failure HR (95%CI) |
| --- | --- | --- | --- | --- | --- | --- | --- |
| 1-2 | Reference | Reference | Reference | Reference | Reference | Reference | Reference |
| 3-4 | 1.01 (0.88, 1.16) | 1.02 (0.87, 1.21) | 0.99 (0.83, 1.19) | 1.00 (0.93, 1.08) | 1.03 (0.93, 1.14) | 1.05 (0.98, 1.12) | **1.02 (0.98, 1.06)** |
| 5-6 | 1.06 (0.91, 1.23) | 1.03 (0.85, 1.23) | 1.06 (0.88, 1.28) | **1.12 (1.04, 1.22)** | 1.09 (0.98, 1.22) | 1.03 (0.96, 1.11) | **1.10 (1.06, 1.15)** |
| 7-8 | 1.11 (0.94, 1.31) | 1.07 (0.87, 1.31) | 0.98 (0.81, 1.20) | **1.09 (1.00, 1.19)** | 1.09 (0.97, 1.24) | 1.02 (0.94, 1.10) | **1.19 (1.13, 1.24)** |
| 9 or more | **1.25 (1.09, 1.45)** | **1.24 (1.05, 1.47)** | 1.02 (0.85, 1.22) | **1.24 (1.15, 1.34)** | 1.08 (0.97, 1.20) | 0.87 (0.81, 1.00) | **1.20 (1.16, 1.25)** |
|  | P = 0.00 | P = 0.01 | P = 0.08 | P = 0.00 | P = 0.02 | P = 0.07 | P = 0.00 |

PUB – perforation or ulceration or bleeding, HR – hazard ratio, CI – confidence interval, P – P_trend_, and significant results are in bold

## References

1. Quan H, Li B, Couris C, et al. Updating and validating the Charlson comorbidity index and score for risk adjustment in hospital discharge abstracts using data from 6 countries. *American Journal of Epidemiology*. 2011;173(6):676-82.
2. Austin P. Balance diagnostics for comparing the distribution of baseline covariates between treatment groups in propensity-score matched samples. *Statistics in Medicine*. 2009;28(25):3083-107.
3. Barbulescu A, Delcoigne B, Askling J, et al. Gastrointestinal perforations in patients with rheumatoid arthritis treated with biological disease-modifying antirheumatic drugs in Sweden: a nationwide cohort study. *Rheumatic and Musculuskeletal Diseases (Open)*. 2020;6(2):e001201.
4. McCaffrey D, Griffin B, Almirall D, et al. A tutorial on propensity score estimation for multiple treatments using generalized boosted models. *Statistics in Medicine*. 2013;32(19):3388-414.
5. Bolch C, Chu H, Jarosek S, et al. Inverse probability of treatment-weighted competing risks analysis: an application on long-term risk of urinary adverse events after prostate cancer treatments. *BMC Medical Research Methodology.* 2017;17(1):1-8.
6. Mitchell J, Gage B, Fergestrom N, et al. Inverse Probability of Treatment Weighting (Propensity Score) using the Military Health System Data Repository and National Death Index. *Journal of Visualized Experiments : JoVE.* 2020(155):10.3791/59825.
